# Bioaccessibility of Phenolic Compounds, Resistant Starch, and Dietary Fibers from Australian Green Banana during In Vitro Digestion and Colonic Fermentation

**DOI:** 10.3390/molecules29071535

**Published:** 2024-03-29

**Authors:** Yasmeen M. Bashmil, Frank R. Dunshea, Rudi Appels, Hafiz A. R. Suleria

**Affiliations:** 1 Department of Food and Nutrition, Faculty of Human Sciences and Design, King Abdulaziz University, Jeddah 21589, Saudi Arabia; ybashmil@student.unimelb.edu.au; 2 School of Agriculture, Food and Ecosystem Sciences, Faculty of Science, The University of Melbourne, Parkville, VIC 3010, Australia; fdunshea@unimelb.edu.au (F.R.D.); rudi.appels@unimelb.edu.au (R.A.); 3 Faculty of Biological Sciences, University of Leeds, Leeds LS2 9JT, UK

**Keywords:** unripe banana polyphenols, RS, DF, digestibility, fermentation, bioavailability, SCFAs

## Abstract

Green bananas contain a substantial amount of resistant starch (RS), dietary fiber (DF), and phytochemicals, which exhibit potent antioxidant capabilities, primarily attributable to the abundance of polyphenols. The objective of this study was to assess the variations in the contents and bioaccessibility of RS, DF, and phenolic compounds in three types of Australian green bananas (Cavendish “*Musa acuminata*”, Ladyfinger “*Musa paradisiaca* L.”, and Ducasse “*Musa balbisiana*”), along with their antioxidant capacities, and the production of short-chain fatty acids (SCFAs) following in vitro simulated gastrointestinal digestion and colonic fermentation. The studied cultivars exhibited significant levels of RS, with Ladyfinger showing the greatest (49%). However, Ducasse bananas had the greatest DF concentration (38.73%). Greater TPC levels for Ladyfinger (2.32 mg GAE/g), as well as TFC and TTC (0.06 mg QE/g and 3.2 mg CE/g, respectively) in Cavendish, together with strong antioxidant capacities (DPPH, 0.89 mg TE/g in Cavendish), have been detected after both intestinal phase and colonic fermentation at 12 and 24 h. The bioaccessibility of most phenolic compounds from bananas was high after gastric and small intestinal digestion. Nevertheless, a significant proportion of kaempferol (31% in Cavendish) remained detectable in the residue after colonic fermentation. The greatest production of SCFAs in all banana cultivars was observed after 24 h of fermentation, except valeric acid, which exhibited the greatest output after 12 h of fermentation. In conclusion, the consumption of whole green bananas may have an advantageous effect on bowel health and offer antioxidant characteristics.

## 1. Introduction

Banana is a tropical fruit that encompasses numerous species within the genus Musa and family Musaceae. It is one of the most popular fruits in the world and the fourth most significant crop produced globally [1]. Green bananas contain far more flavonoids, dietary fiber (DF), and resistant starch (RS) than mature bananas [2]. Bananas have a higher antioxidant capacity than many cereals, herbs, and vegetables due to the presence of phenolics, carotenes, flavonoids, and other phytochemicals [3]. The biological effects and health benefits of phenolic substances are well known. Nevertheless, the bioavailability and stability of these compounds during digestion and assimilation have a significant impact on their health benefits.

Bioaccessibility and bioactivity are crucial variables for bioactive compounds, which are typically employed to evaluate the primary nutritional efficacy of living beings [4]. In vitro digestion can be utilized to simulate physiological conditions and assess the bioaccessibility of bioactive compounds and nutrients after food ingestion [5]. The impact of in vitro digestion on the amounts of phenolic compounds and antioxidant capability in different fruits and vegetables has been assessed in recent research [6]. Nevertheless, the existing body of knowledge regarding the in vitro digestion and colonic fermentation of green banana is extremely limited. Several previous studies on the persistence along the gastrointestinal tract as well as the availability of dietary phenolics following gastrointestinal digestion demonstrated that these factors varied greatly from one polyphenol to another, dependent on the amount released from the dietary matrix [7,8].

Complex carbohydrates consist of starch and non-starch polysaccharides with widely varying molecular structures and physiological impacts. Resistant starches are defined as “some starches and byproducts of starch degradation that are not absorbed in the small intestine of healthy individuals” [9]. Dietary fibers, however, are defined as “Carbohydrate polymers with ten or more monomeric units that are impermeable to human-produced digestive enzymes” [10]. A widely accepted categorization of dietary fiber comprises the following: soluble dietary fiber, comprising hydrocolloids, oligosaccharides, and non-cellulosic polysaccharides such as pectin, β-glucan, and gums; and insoluble dietary fiber, which include resistant starch, cellulose, hemicellulose, and lignin [11].

A crucial characteristic of RS is that it is not digested or broken down within a two-hour period in the intestine, and, thus, it promotes the proliferation of probiotic bacteria in the colon, which may have an indirect beneficial influence on reducing the risk of colon cancer [12]. Whole green banana, including the skin, is an abundant source of RS (41–59%) [13], and the flour made from green bananas has been suggested to add value to banana crops by minimizing the amount of waste produced during the production chain, improving sustainability, and capturing some of the beneficial nutrients that vanish throughout maturation [14].

Starch digestion begins in the mouth, where salivary amylase breaks down starch into saccharides and glucose molecules, which are then absorbed directly from the intestinal mucosa located in the small intestine [15]. However, in the large bowel, colonic microflorae ferment undigested starch to produce short-chain fatty acids (SCFAs) that promote the proliferation of beneficial gut bacteria [1]. The effect of in vitro digestion on Australian banana polyphenols, RS, and dietary fibers and the resultant short-chain fatty acids after gut fermentation has not been studied previously. Thus, the objective of this study was to determine the antioxidant potential and bioaccessibility of a variety of phenolic compounds, resistant starch, and dietary fibers from banana flour obtained from green Australian-grown Cavendish, Ladyfinger, and Ducasse bananas using a static in vitro approach of gastrointestinal digestion and colonic fermentation.

## 2. Results and Discussion

### 2.1. In Vitro Digestion of Resistant and Non-Resistant Starch and Dietary Fibers in Whole Green Banana

The total dietary fiber (TDF) and resistant starch contents of the green banana are displayed in Table 1. The resistant starch concentration varied from 49.0 ± 0.21% to 31.5 ± 0.17%, with Ladyfinger banana having the greatest value and Cavendish the lowest. The Ladyfinger variety contained significantly more resistant starch than the other two cultivars (Cavendish and Ducasse) (Table 1). The non-resistant starch level ranged from 11.89 ± 0.18% to 23.39 ± 0.13% in Ladyfinger banana and Cavendish, respectively. Ladyfinger, which had the highest value of RS, had the lowest amount of nonresistant starch. Similarly, Udo et al. [16] discovered that the Ladyfinger banana had the highest RS content compared to the plantain variety, Akpakpa (French Horn) banana. In addition, a significant positive correlation was previously observed between amylose concentration and resistant starch content. The percentages of resistant starch observed in this study were higher than those previously published for immature banana (38.3 ± 3.10%) [17] and KluaiKhai banana (35.14 ± 2.41%) [18]. The variations noticed during this investigation may be attributable to distinctions in cultivar and digestion technique.

Moreover, Table 1 displays the amount of TDF in the studied samples. Ducasse bananas had the highest level of dietary fiber (38.73 ± 0.27%), while Cavendish and Ladyfinger bananas had the lowest amount (31.29 ± 0.39% and 31.02 ± 0.68%, respectively). In contrast to Ladyfinger, the Ducasse sample had a very high dietary fiber content, which compensated for its apparent lack of RS. According to a previous study, the TDF found in green banana flour was 14.52%, which is significantly less than the RS level obtained in this research for three types of unripe banana powder [18]. However, some reports suggested that other kinds of banana have less TDF content (6.28%) and (9.2%) [19,20].

It appears that Australian banana starch was extremely resistant to enzymatic hydrolysis. Multiple factors have been suggested to explain the resistance of some starches, including the degree and type of crystallinity [21], amylose content [22,23], granular size [24], and morphology and structure, such as granule surface area and porosity [25,26]. Additionally, the remaining cell walls in banana flour might have protected starch granules from enzyme degradation by entrapping them.

As stated previously [20], additional amylase hydrolysis of in vivo samples revealed that the starch particles had gone through structural changes, making them more susceptible to breakdown. A significant portion of the starch accumulated at the end of the ileum might have been metabolized by amylolytic enzymes. Traveling through the small intestine may have increased the accessibility of certain structures by modifying the surface area and porosity of the granules without affecting their structural integrity. The outcome of RS upon entrance into the colon is largely determined by its structure. Oligosaccharides and the readily digestible portion of RS must ferment faster than the resistant portion. Most of the starch that entered the colon was metabolized by microflorae, according to investigations regarding overall digestibility [20,27,28].

Notable is the fact that the type of green banana resistant starch, which is composed of the native resistant starch granules only ingested as unripe banana (RS2), diminishes with ripening due to the conversion by the endogenous amylase of RS2 into reducing sugar [29]. Thus, drying will be important to inactivate RS2 in banana flours and preserve it therein. The influence of oven and freeze-drying on banana flour dietary fibers’ quantity has been investigated [30], and it has been found that drying in the oven leads to a percentage of 26.8% insoluble dietary fibers, while freeze-drying produces a concentration of 43.2% undissolved fibers.

### 2.2. Phenolic and Bioactivity Changes during In Vitro Digestion

#### 2.2.1. Effects of In Vitro Digestion on TPC, TFC, and TTC

**Figure 1:**
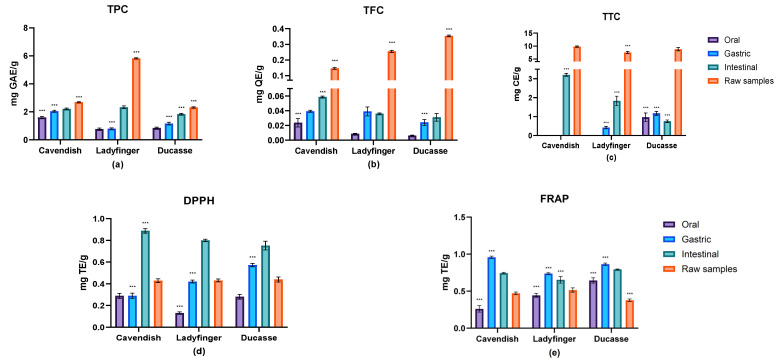
The assessment of the phenolic content of green banana in vitro digestates. (**a**) Total phenolic content; (**b**) total flavonoids content; (**c**) total tannins; (**d**) 2,2′-diphenyl-1-picrylhydrazyl antioxidant assay; (**e**) ferric reducing antioxidant power assay. The findings of all assays are presented as mean ± standard deviation (*n* = 3) on a dry weight basis with the control values subtracted. Orange bars: raw samples; purple bars: oral phase; blue bars: gastric phase; green bars: intestinal phase. GAE: gallic acid equivalents; QE: quercetin equivalents; CE: catechin equivalents; TE: Trolox equivalents; ***: statistically very significantly different (*p* ≤ 0.01).

Figure 1 displays the results of the total phenolics, total flavonoids, and total hydrolyzable tannins of the raw and digested samples of the three green banana powders (Cavendish, Ladyfinger, and Ducasse). The findings indicated that the TPC, TFC, and TTC of all banana samples declined significantly after in vitro digestion compared to the raw samples. Each stage of digestion influenced the phenolic content, particularly for Ladyfinger, in which the oral, gastric, and intestinal phases diminished the TPC by 87%, 87%, and 60%, respectively, in comparison to the indigestible samples (Figure 1). However, the phenolic content of the samples digested during the oral and gastric phases did not differ significantly.

The oral, gastric, and intestinal reductions in TPC were 40.5%, 25.3%, and 18.9% for Cavendish samples, respectively. Similarly, Ladyfinger and Ducasse bananas showed a significant drop during all digestion stages compared to the raw samples. In all cultivars, Cavendish, Ladyfinger, and Ducasse, a greater amount of TPC was released during the intestinal phase of digestion compared to other stages of digestion. It was noted that intestinal enzymes might work on dietary residuals to permit more release of phenolic compounds and increase their total quantity [31,32]. Furthermore, another study reported that about 30 to 48% of chlorogenic acids could be metabolized in the small intestine. The remaining fragments might break down more in the large intestine [29]. The related hydroxycinnamates, such as caffeic, ferulic, and quinic acids, might be produced by chlorogenic acids’ degradation and ultimately increase the TPC value [33]. As a result, during the intestinal digestion stage, the hydrophobic bonds between carbohydrates and phenolic compounds may be diminished in an acidic pH (lower than 6.9), and in the presence of α-amylase, pancreatin, lipase, and bile salts, which facilitate the transport and bioaccessibility of phenolic compounds [34]. In addition, it is worth noting that the presence of bacteria within the digestive system also holds significant importance [35].

No studies have examined the influence of in vitro digestion on bananas’ phenolic acids. However, compared to other plants, Sancho et al. [36] found that the phenolic content of the metabolized extract of small red and black beans decreased by 52% and 75%, respectively. Similarly, another study discovered a significant decrease in the TPC of araticum (Annona marcgravii) pulp extract (from 215.70 to 178.20 mg GAE/g after digestion) and papaya pulp yield (from 79.50 to 28.6 mg GAE/g after digestion) [37].

Considering the TFC content, all metabolized banana cultivars exhibited a substantial reduction compared to the raw samples, as shown in Figure 1. The Ducasse extract considerably decreased (*p* < 0.01) the level of flavonoids during the digestive phases (−98.25% for the oral phase, −93.2% for the gastric phase, and the least flavonoids loss (−91.23%) in the intestine). In a similar way, in other banana samples, the TFC diminished significantly through the in vitro digestion procedure, particularly during the oral phase. This was comparable to what was observed by Chait et al. [38], when they studied the simulated gastrointestinal digestion of carob polyphenols. They observed little change in carob polyphenols during the oral phase in comparison to the other digestion stages, which could potentially be attributed to the limited contact time through the oral phase (2 min) as well as the relatively low activity (1.25 µkat/mL) of α-amylase responsible for initiating starch hydrolysis in the mouth. Similarly, de Paulo Farias et al. noticed that the TFC content in uvaia seed extract decreased substantially during the digestive phases (−48% for the gastric and −70% for the intestinal phases) [39].

On the contrary, the intestinal TFC was significantly higher than the oral and gastric phases, which could be attributed to the possibility that intestinal digestion could dramatically improve the release of flavonoids from the food matrix. Our results concurred with Wu et al. [40], whose research demonstrated a sudden increase in the TFC content of light- and dark-roasted coffee beans after intestinal digestion.

Low levels of TPC and TFC in the oral phase (after 2 min of digestion) can be attributed to the limited solubility of these compounds in saliva and the brief duration of this step. According to Ortega et al. [41], physicochemical changes, such as oxidation or interactions with other substances, including polysaccharides in the digestion mix, might clarify the phenolic depletion during in vitro digestion. In addition, a reduction in phenolic compounds may result from the precipitation of certain phenolics, such as tannins, with proteins (enzymes) during digestion. In this context, González-Sarrías et al. [42] stated that the pH and protein environment restricted the absorption of ellagic acid and ellagitannins.

In the current research, TTC content digestion in all banana samples followed a distinct pattern (Figure 1). The amount of tannins in these samples decreased over all digestion phases; however, in the Cavendish and Ladyfinger samples, findings during oral digestion were undetectable. Tannin metabolites are degraded more by gut bacteria due to their complicated composition [43]. On average, the hydrolyzable tannins and high-molecular-weight proanthocyanidins constitute more than 75% of the total polyphenolic compounds in food. These molecules can bind closely to dietary fibers, restricting their availability [44]. Similar to our findings, Quatrina et al.’s [45] experiment on Jaboticaba fruits showed that the hydrolyzable tannins had the largest decrease after salivary digestion (>74% decrease). In addition, more than 80–90% of these hydrolyzable tannins were degraded after gastric and intestinal digestion. Spencer et al. [46] revealed that the procyanidins oligomers (trimer to hexamer) might become degraded and hydrolyzed into epicatechin monomer and dimer in the acidic gastric medium. Nevertheless, Hollman [43] stated that only proanthocyanidins composed of fewer than three catechins were likely absorbed from the intestine. According to Koleckar et al. [47], only original monomers and dimers can be ingested in the stomach. On the other hand, the mild alkaline circumstance might stimulate the hydrophobic interactions between protein and tannins, leading to precipitation [32].

#### 2.2.2. Antioxidant Activities Estimation

The antioxidant activity throughout the various in vitro phases was assessed using the 2,2′-diphenyl-2-picryl-hydrazyl (DPPH) and ferric reducing antioxidant power (FRAP) assays in this study. The DPPH assay relies on an electron transfer mechanism and quantifies the suppression of nitrogen free radicals. In contrast, the FRAP assay differs from this approach as it does not involve the presence of free radicals. The methodology employed in this assay consists of the observation and measurement of the reduction of ferric iron (Fe^3+^) to ferrous iron (Fe^2+^) [48]. The antioxidant properties of banana samples that went through in vitro digestion are demonstrated in Figure 1. Cavendish exhibited comparatively elevated efficacy in scavenging free radicals and in ferric ions’ reducing antioxidant potential during the process of in vitro digestion.

Based on DPPH results (Figure 1), the capacity of all samples to scavenge free radicals expanded significantly after oral consumption, with some differences between cultivars, which is likely due to the presence of some bioactive compounds, particularly for specific varieties, that exhibit limited solubility in the DPPH reaction medium. As a result, the radical scavenging activity is hindered, leading to a decreased performance in terms of radical scavenging [49]. Furthermore, the findings of this study suggest that the phenolic compounds responsible for the antioxidant activity of bananas are primarily released during the simulated intestinal phase. This aligns with previous research on plant seeds showing lower antioxidant capacity during the oral and gastric phases. In contrast, apples and hawthorn fruits exhibit high antioxidant activity during these phases [50,51], mainly attributed to the presence of phenolic compounds in food materials, which are released in different forms and ratios at various stages of digestion [52].

On the other hand, the associated FRAP was substantially greater in samples digested in the stomach, especially in Cavendish, with about 0.96 mg TE/g (Figure 1). The overall decrease in FRAP values was noted throughout intestinal digestion, as demonstrated by earlier in vitro digestibility studies of apples [53] and lettuce [54]. Whereas the small intestine is considered the primary location for absorption of free phenolics, an alteration in the pH condition during intestinal metabolism impacted the volume of released phenolic compounds. There was a greater probability that part of the phenolic compounds, which were sensitive to the mildly alkaline pH levels of the small intestine, were converted into structural types with distinct chemical attributes, thereby altering their biological activity [55]. In opposition to the phenolic content, the antioxidant properties of the three banana cultivars increased substantially during gastric and intestinal digestion. The steady rise in DPPH values results from the continued release and accumulation of phenolic compounds from bananas.

Banana peel and pulp contained significant quantities of cell wall-bound phenolics, including anthocyanidins, quercetin, and cyanidin-3-*O*-glucoside chloride [56,57,58]. Because bound phenolics are attached to unresolved macromolecules, their routes of transport and absorption mechanisms in the digestive system vary from those of free phenolics. Free phenolics go through limited release in the mouth and absorption in the small intestine. In contrast, bound phenolics migrate right away to the bowel, where they are fermented by gut microbiota, causing the release of the bound phenolics [59]. Despite this, free and bound parts are providers of naturally occurring antioxidant compounds, as demonstrated by various antioxidant experiments conducted in this study.

Additionally, the high antioxidant capacity discovered in this study suggests that phenolic compounds were not the only antioxidant producers within the fruit crop. Notably, other secondary metabolites with antioxidant abilities can be responsible for the increased antioxidant activity of the Saba banana, including vitamin C, β-carotene, and vitamin E [60]. Mainly during the gastric phase, antioxidant capacity increased. These compounds are robust in acidic environments [61,62]. This was confirmed by previous in vitro research that showed high flavonoids and phenolic acids stabilization and rate of release in stomach circumstances [55,63].

Another illustration is the biochemical change in the anthocyanidin into less stable compounds even at nearly neutral pH [64], which ultimately results in anthocyanin breakdown, leading to anthocyanin degradation [55,65]. Similarly, it has been found that flavanols, for example, quercetin, endure oxidation, hydroxylation, and ring breakage at medium pH levels in a manner influenced by time, leading to the development of complex substance profiles [66]. A further potential explanation that might clarify the patterns is that the antioxidant activity results were influenced by the tests’ sensitivity to pH variations, which might additionally account for the unexpectedly elevated DPPH and FRAP values throughout the gastric and intestinal phases.

### 2.3. Phenolic and Bioactivity Changes during Colonic Fermentation

#### 2.3.1. Phenolic Evaluation

**Figure 2:**
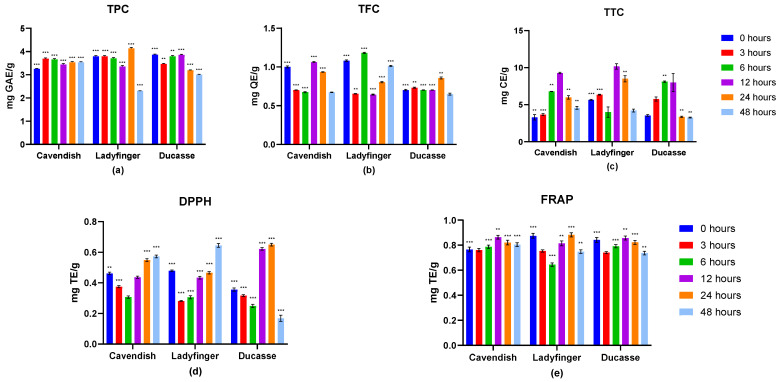
The antioxidant capacity of green banana digesta following colonic fermentation. (**a**) Total phenolic content; (**b**) total flavonoids content; (**c**) total condensed tannins; (**d**) 2,2′-diphenyl-1-picrylhydrazyl antioxidant assay; (**e**) ferric reducing antioxidant power assay. The assays’ findings were reported as the mean ± standard deviation (*n* = 3) based on dry weight after subtracting the control values. Dark blue bars: 0 h; red bars: 3 h; green bars: 6 h; purple bars: 12 h; orange bars: 24 h; light blue bars: 48 h. GAE: gallic acid equivalents; QE: quercetin equivalents; CE: catechin equivalents; TE: Trolox equivalents; **: statistically significantly different (*p* ≤ 0.05); ***: statistically very significantly different (*p* ≤ 0.01).

The changes in total phenolic compounds, total flavonoids, and hydrolyzed tannin contents in banana cultivars after colonic fermentation are illustrated in Figure 2. Following fermentation for 3 h, the Cavendish TPC value displayed a notable rise of 3.69 mg GAE/g. This suggests that there is a favorable effect resulting from the production of phenolic compounds by colonic fermentation. However, Ladyfinger banana showed a stable TPC content during 0 and 3 h and reached the highest point at 24 h with 4.14 mg GAE/g. At the same time, Ducasse TPC content decreased to some degree, to about 3.47 mg GAE/g after 3 h fermentation. Both Ladyfinger and Ducasse cultivars had the lowest phenolic content after 48 h.

According to Acosta-Estrada et al. [38], undigested polyphenolic compounds, particularly those bound covalently to food matrix molecules, resist digestive enzymes during colon transit. In the colon, these compounds can undergo catabolism through the action of existing microflorae, resulting in their degradation into phenolic acids through enzymatic release [29]. The observed influence on TPC at the early stage of colonic fermentation in our study aligns with the findings of Luo et al. [67]. Their research demonstrated a considerable increase in TPC within a 2 h timeframe, particularly in relation to black and brown sesame seeds. Additionally, it was found that the phenolic content of the three sesame seed varieties reached its maximum level after 8 h of fecal reaction. Among the cultivars studied, white sesame seeds exhibited the highest colonic TPC value at 4.17 mg GAE/g, followed closely by brown sesame seeds with a TPC value of 4.13 mg GAE/g. These findings correspond strongly with our data.

As for total flavonoids in this phase, the three banana cultivars had different performances. Cavendish and Ladyfinger flavonoids decreased considerably after 3 h, but they considerably increased again to reach the greatest amount after 6 h (1.18 mg QE/g) and 12 h (1.06 mg QE/g) for Ladyfinger and Cavendish, respectively. However, TFC results of Ducasse banana peaked after colonic fermentation for 24 h, with the least flavonoid content (0.85 mg QE/g) compared to the other two cultivars. The reported increase in TPC and TFC indicates that the process of colonic fermentation could involve the additional release of phenolic compounds from bananas, enhanced by the activity of gut microbiota.

Comparing all three green banana cultivars in our study, the release of phenolic compounds is contingent upon the metabolic processes of bacteria during gut fermentation. The potential cause for the observed variation in flavonoid concentrations across the three banana varieties could be attributed to changes in their original flavonoid levels. Moreover, the presence of complex carbohydrates in plant tissue, such as dietary fiber, may directly impact the interaction with antioxidant compounds, potentially hindering their optimal release and digestion [68]. Parada and Aguilera [69] also suggested that the food microstructure has a significant role in the release and absorption of various bioactive compounds, particularly those with exceptional antioxidant properties. According to Brownlee et al. [70], including dietary fiber in the small intestine can lead to the extended release of bioactive compounds and other nutrients. This is achieved through the physical entrapment of these compounds within the fiber’s structure and the restriction of enzyme transport due to the increased viscosity of gastric fluids.

TTC in banana digesta generally showed growing trends in the three tested banana digesta (Figure 2). The TTC values of Cavendish and Ladyfinger reached the highest point after fermentation for 12 h at 9.27 mg CE/g and 10.16 mg CE/g for Cavendish and Ladyfinger, respectively. In contrast, Ducasse tannins climbed to 8.10 mg CE/g following 6 h and retained this growth for 12 h of fermentation. Our results indicate that Ladyfinger may have the greatest condensed tannin content that is released after microbial fermentation in the large intestine. Previous research established that trace quantities of condensed tannins have been identified in the residual material after fermentation. This finding indicates that most condensed tannins are liberated from the food matrix and rendered soluble in the fermentation media by the enzymatic activity of colonic bacteria. Additionally, their outcomes indicated that a significant proportion of proanthocyanidins, around 95%, were liberated from the food matrix by the enzymatic activity of bacteria, and approximately 46% of dietary tannins were able to be accessed by the body in the large intestine [4,71].

#### 2.3.2. Antioxidant Capacity Estimation

The antioxidant capacities of three banana samples changed, as shown in Figure 2. Generally, the DPPH of banana digesta decreased significantly at 3 and 6 h of fermentation, but after that, the results showed a considerable improvement by colonic fermentation. A similar variation was noticed in all studied banana cultivars, with values significantly dropping from about 0.46, 0.47, and 0.35 mg TE/g (0 h) to 0.31, 0.30, and 0.24 mg TE/g after 6 h of fermentation for Cavendish, Ladyfinger, and Ducasse, respectively, then suddenly climbing back after 12 h. The Cavendish and Ladyfinger DPPH levels exhibited a notable increase again, ultimately reaching a peak of about 0.57 and 0.64 mg TE/g at 48 h of fermentation. However, Ducasse reached its highest point after 24 h. This indicated a correlation between the free radical scavenging capability of bananas and their phenolic component concentration, particularly flavonoids. Similarly, banana samples had the greatest FRAP rates during fermentation periods of 12 and 24 h, especially Ladyfinger with 0.88 mg TE/g, and this finding corresponded with Ladyfinger’s total phenolics content results at 24 h. These findings provided clear evidence that bowel fermentation plays a significant role in assisting the release and degradation of phenolic compounds present in the remaining banana digesta and enhancing its in vitro antioxidant activity. It may be considered that there is a possible connection between the lower FRAP value during certain fermentation stages and a decreased initial concentration of phenolic compounds and other bioactive compounds, including vitamin C, which possess potent antioxidant properties [71].

### 2.4. Bioaccessibility of Individual Phenolic Compounds in Green Banana

The biological accessibility of fifteen phenolic compounds throughout various digestive phases and fermentation stages is presented in Table 2. The term “bioaccessibility” refers to the proportion of bioactive substances that are consumed and have the potential to be absorbed by the epithelial layer of the gastrointestinal tract [4]. Upon conducting a comprehensive analysis, it was revealed that the gastric and intestinal phases exhibited the maximum bioaccessibility for most phenolic chemicals. The bioaccessibility of total phenolic compounds was shown to be at its lowest level following colonic fermentation, which aligns with the findings published by de Almeida et al. [72]. Their study also observed a continuous decline in total phenolic bioaccessibility, which reached its lowest point after 24 h of fermentation. The results of Konishi et al. [73] and Saura-Calixto et al. [74] support the notion that phenolic acids, which are the primary phenolic chemicals found in vegetables and fruits, are typically absorbed in their aglycone form in the upper section of the digestive tract. As found in previous research, the stomach and small intestine could be the primary sites for the active absorption of phenolic acids, including gallic acids and caffeic acids [73]. Hence, it can be inferred that most phenolic acids have the potential to be liberated from bananas because of gastrointestinal digestion, thereby agreeing with the results obtained in our study.

Moreover, our study also revealed variations in the bioaccessible site and bioaccessibility of the same phenolic compounds among different banana varieties. For example, the release of protocatechuic acid from Ducasse was significantly higher during the gastric phase. At the same time, its bioaccessibility was greater during the intestinal stage following the ingestion of the Cavendish and Ladyfinger varieties. Tarko and Duda-Chodak [75] also verified that the discrepancies in the bioaccessibility of phenols mostly arise from the interactions between phenols and the food matrix. The extractability and sensitivity to digestive enzymes and bacterial metabolism may be altered when phenolic chemicals bind to a food matrix with various compositions [76]. Along with the structure of the dietary fiber, Rodríguez-Roque et al. [77] also discovered that the mineral content of the fruit juice may contribute to the interactions with phenolic compounds and subsequently limit the release of phenolic compounds. Additionally, it has been suggested that the inclusion of sugar may enhance the bioaccessibility of phenolic compounds through mechanisms such as decreasing interactions between tannins and pepsin or enhancing the solubility of tannin–pepsin complexes, which strongly justifies the observed considerable polyphenols’ release from banana during gastric and intestinal stages in this study.

As shown in Table 2, chlorogenic acid’s bioaccessibility was the highest throughout salivary digestion, especially of Cavendish (33.24%), followed by that of protocatechuic and hydroxybenzoic acid. In theory, it has been suggested that the body cannot readily absorb approximately 60% of chlorogenic acid present in bananas. Instead, it requires further enzymatic breakdown by gut microbiota during the process of colonic fermentation to liberate caffeic acid. This is believed to be due to the absence of esterase enzymes [78]. In a study conducted by Olthof, Hollman, and Katan [79], it was observed that the bioaccessibility of chlorogenic acid from the gastrointestinal tract, particularly the small intestine, was limited to a maximum of 33% of the ingested amount. Notably, all banana phenolic compounds showed a significant rise after gastric and intestinal digestion, which was consistent with the results of Ordoñez-Díaz et al.’s [80] study, when they found that mango’s total polyphenols concentration was higher after in vitro gastric digestion than the initial quantities. The noted rise can be elucidated by considering the release of polyphenols during the gastric phase. These polyphenols, which exist as hydrogen bonding structures or are covalently attached to cell wall polysaccharides within the food matrix, are liberated due to the acidic environment and the presence of pepsin [81]. Consistent with our findings, Lucas-González et al. [82] observed a statistically significant rise in the concentration of gallic acid in persimmon fruits following gastric digestion.

Following the in vitro intestinal digestion, there was a considerable increase in the overall polyphenol content within the banana. The observed rise can be primarily caused by a substantial increase in the concentration of certain phenolic compounds during the intestinal digestion, such as gallic acid, from 27.63% to 79.11% for Cavendish, protocatechuic acid (from 32.67% to 61.26%) for Ladyfinger, hydroxybenzoic acid (from 28.87% to 45.82%) for Cavendish, and chlorogenic acid, which climbed from 30.79% to 55.50% for Ducasse banana. At the intestinal level, various factors such as pH, the enzymatic activity of pancreatin, and the presence of bile salts may contribute to the disruption of weak bonds between supramolecular structures within the food matrix and polyphenols. This phenomenon is particularly significant for polyphenols with lower molecular weights, as it facilitates their release from the food matrix during the process of digestion and enhances the bioaccessibility of these polyphenols [83]. All these substances are possibly absorbed after the completion of intestinal digestion.

Upon investigating the effect of digestion on flavonoids’ bioaccessibility, our investigation revealed that the intestinal phase had several advantages compared to gastric treatment, notably resulting in elevated levels of flavonoids. The potential interaction between flavonoids and protease at a low pH could lead to the formation of flavonoid–protease complexes. In accordance with a previous study, the binding capacity between digestive enzymes and catechins could be influenced by the pH values in the gastric or intestinal environment [84]. The interaction between flavonoids and pepsin resulted in a reduction in the solubility of flavonoids in acidic solutions. The release of phenolic compounds was enabled by trypsin, which caused the liberation of protein-bound phenolics, and the reduction in chyme’s particle size was found to have a positive effect on the release of phenolic compounds. In addition, several phenolic compounds were liberated from the matrix under nearly neutral pH conditions, but through the stomach phase.

Another potential factor might be physical entrapment by dietary fiber. A possible interaction between dietary fiber and phenolic compounds arises from hydrophobic aromatic rings and hydrophilic hydroxyl groups in phenolic compounds [40]. The linkage between phenolic compounds and polysaccharides is established through hydrogen bonding, specifically between the hydroxyl group of phenolic compounds and the oxygen atoms within the glycosidic linkages of polysaccharides. Further, hydrophobic interactions and covalent bonds, such as ester bonds, are also present. The hydrolysis of these bonds necessitates the participation of bacterial enzymes [68].

This study showed that the main flavonoid compounds in the green banana were catechin, its isomer (epicatechin), and quercetin. Catechin and epicatechin are the building blocks of condensed tannins, which could probably be produced during the digestion of tannins. The data showed these flavonoids markedly increased after intestinal digestion in the three banana varieties. Also, gallic acid, as a precursor molecule for tannins, can be produced through the hydrolysis of chlorogenic acids and tannins. Throughout the entire process of digestion, gallic acid consistently demonstrates notable bioaccessibility [85].

### 2.5. Recovery and Residual Index of Individual Phenolic Compounds in Green Banana

The recovery and residual index of each of the unique phenolic compounds identified in bananas are displayed in Table 3. Cavendish and Ducasse showed a comparable recovery index of total phenolic compounds, yielding a much lower result than Ladyfinger, in which the total phenolics recovery was 32.05%. Almost all phenolic compounds found in bananas could be liberated entirely to become bioaccessible through the procedure of colonic fermentation. The outcomes of this study provide compelling evidence that colonic fermentation could promote the release and breakdown of phenolic compounds derived from the indigestible component of bananas.

In general, most banana phenolic compounds decreased considerably after colonic fermentation. For example, the intestinal availability of gallic acid in Cavendish and Ladyfinger bananas were about 79% and 69%, respectively, whereas their colonic recovery indexes were directly reduced to 12% and 17%. In banana samples, the complete release of gallic acid occurs during intestinal digestion, followed by its continuous degradation throughout the fecal reactions by gut bacteria, with some detected in the residual digesta. Nevertheless, the residual colonic digesta index (RCDI%) values of quercetin in all three cultivars and kaempferol in Cavendish and Ducasse still performed with a high percentage, similar to the amounts found after intestinal digestion.

For gallic acid, its presence in the colonic residue is associated with the subsequent breakdown of several complex phenolic compounds, including chlorogenic acids. This degradation could lead to the continuous production of gallic acid, which then combines with the remaining components in the substrate to form precipitates. The colonic recovery index of gallic acid also indicates that a significant proportion of intact gallic acid can be recovered without undergoing breakdown. Additionally, it can be concluded that 48 h of colonic fermentation may not be sufficient for the comprehensive absorption of phenolic compounds in green banana. Interestingly, green bananas evidenced a great recovery of quercetin after 48 h of fermentation. These findings were supported by Attri et al. [86] when they found that the digested fraction of sea buckthorn berries’ juice resulted in the highest quercetin level after 36 h. This reported elevation of quercetin levels could be related to the conversion of various polyphenolic compounds into quercetin by gut microbiota, specifically *Bacteroides uniformis* and *Bacteroides ovatus*. These particular bacteria can metabolize rutin and convert it into quercetin [33].

Similarly, kaempferol was highly recovered after colonic fermentation in the Cavendish and Ducasse bananas. According to previous research, kaempferol would commonly conjugate with other components, such as dietary fiber, through an *O*-glycosidic bond, which needs further enzymatic activity by gut microorganisms to become bioaccessible [87]. Furthermore, the study conducted by Cárdenas-Castro et al. [35] proposed that the metabolism of kaempferol could lead to the production of *p*-hydroxybenzoic acid. This finding concurs with our research outcomes.

On the other hand, diosmin exhibited a complete absence of colonic recovery and residual levels in all analyzed banana samples. In accordance with Silvestro et al. [88], it has been discovered that diosmin could undergo significant conversion to aglycone diosmetin through the action of gut bacteria upon consumption. This reaction resulted in the formation of bioaccessible metabolized diosmin, which was in agreement with our results.

### 2.6. Short-Chain Fatty Acids (SCFAs)

**Figure 3:**
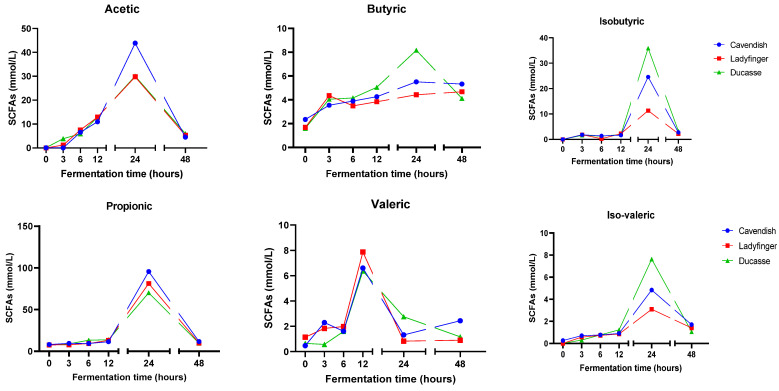
The pattern of generation of SCFAs in green banana digesta after complete colonic fermentation.

The production pattern of six short-chain fatty acids (acetic, butyric, iso-butyric, propionic, valeric, and iso-valeric) in three fermented banana varieties are illustrated in Figure 3. Mainly, propionic acids were the most prevalent SCFAs in the fermentation of all banana cultivars, followed by acetic acid and iso-butyric acid, which is inconsistent with an earlier study conducted by Shi et al. [89], in which acetic acid was the most abundant SCFA in different fermented lettuce varieties. A possible cause for this dissimilarity could be that a various type of dietary fiber caused this case. Dietary fiber could influence the quantity and type of SCFAs generated within the colon [90]. A former study by Welli et al. [91] confirmed our results when dietary intervention with Cavendish banana flour showed a high propionic acid production.

Following the process of gastrointestinal digestion, bananas were proven to retain substrates beyond polyphenols that can be metabolized by gut bacteria. This is related to their significant quantities of dietary fiber and non-fibrous carbohydrates, as shown previously in Table 1. Carbohydrate fermentation serves as the primary energy source for gut microbiota, forming SCFAs. These acids correlate with reduced pH levels and gas generation [92]. Similarly, new findings obtained by in vitro colonic fermentation of an extract rich in tannins support the notion that tannins can serve as a further substrate to produce SCFAs. Indeed, previous studies have demonstrated that tannins exhibit more substantial influence than inulin in the production of acetic, butyric, and propionic acids, with this result demonstrated through in vitro fermentation experiments using human feces [93].

The fermented Cavendish exhibited the greatest production of propionic acids (almost 95.5 mmol/L) followed by Ladyfinger and Ducasse. Distinct variations in the synthesis of SCFAs were detected among diverse banana cultivars. All SCFAs produced through the fermentation of banana, excluding valeric acid, reached their highest amounts at 24 h of colonic fermentation. However, Cavendish and Ducasse generated the greatest contents of most of the studied SCFAs. The fermented Ladyfinger exhibited the most increased production of valeric acid after fecal reactions for 12 h. The pattern of synthesis of SCFAs of all banana varieties was comparable, especially after 12 h of fermentation.

The production of short-chain fatty acids through the microbial fermentation of dietary fibers mainly encompasses acetate, propionate, and butyrate. These compounds have significant implications in regulating energy metabolism, immunological function, and gut cell proliferation in the body [94]. The SCFAs might be able to preserve the proper functioning of the large intestine and safeguard it against pathological conditions by creating an acidic environment in the colon, with particular emphasis on butyric acid [95]. The formation of SCFAs is primarily associated with the breakdown of carbohydrates, particularly resistant starch and dietary fiber, and specific bacterial species in the large intestine [96,97]. As stated by Wong et al. [96], the production of SCFAs can be influenced by both the substrate source and the colon’s passage time. As the main indigestible dietary fibers in green banana, the cellulose, hemicelluloses, and lignin contents in Cavendish and Ducasse could be higher than those of Ladyfinger, which agreed with the formerly revealed TDF results in this study. Nonetheless, our findings showed a significant drop in SCFA content in all banana samples after 48 h of colonic fermentation. That could result from the depletion of dietary fiber as fuel for gut bacteria, or the reduced microbiota diversity at the distal colon [98].

## 3. Materials and Methods

### 3.1. Production of Green Banana Flour

Banana flour was made from Australian-grown green bananas (Cavendish, Ladyfinger, and Ducasse), including banana pulp and skin, which were purchased from different local markets in Melbourne. The green bananas were rinsed with distilled water and, to prevent browning caused by enzymes, the green bananas were sliced to a width of two millimeters, immersed in 0.5% (*w*/*v*) citric acid, and then drained. After four hours in a −80 °C freezer, banana samples were moved to a freeze-dryer (Zirbus VaCo5 System, Bad Grund, Germany) at −50 °C and a pressure of 0.5 hPa for 72 h. The dried green banana pieces were subsequently ground using a coffee grinder (Breville Smart Grinder TM Pro, model BCG820BSSXL, Melbourne, VIC, Australia) to obtain banana powder with a mean particle size of 200 µm, then packaged in plastic containers, and kept at 4 °C until evaluations.

### 3.2. In Vitro Digestion of Resistant Starch (RS) and Non-Resistant Starch (Non-RS) 

The official method used to measure RS was AACC 32-40.01/AOAC 2002.02. A Megazyme Kit was used to carry out the procedure (Megazyme International Ireland Ltd., Wicklow, Ireland). In this procedure, samples (100 mg) were mixed with sodium maleate buffer (pH 6.0) containing pancreatic α-amylase and amyloglucosidase (AMG). Then, samples were incubated in a shaking water bath at 37 °C for 16 h; in the meanwhile, non-RS was hydrolyzed to d-glucose by the action of the two enzymes. This reaction was ceased by adding ethanol (99% *v*/*v*). The resultant mixture was centrifuged for 10 min at 3000 rpm after being rinsed twice with aqueous ethanol (50% *v*/*v*). The supernatant was removed, and by vigorously stirring for 20 min, the pellet (RS) was dissolved in 2 M KOH in an ice/water bath. The solution was neutralized using sodium acetate buffer, and the RS was hydrolyzed to glucose with AMG (water bath for 30 min at 50 °C). After sample centrifugation, an aliquot was mixed with glucose oxidase/peroxidase reagent (GOPOD) and incubated at 50 °C for 20 min. Eventually, the absorbance of each solution was compared to that of the reference standard (blank solution) at 510 nm. On the other hand, the non-resistant starch was estimated by combining all supernatant solutions obtained after the initial incubation and adjusting the volume to 100 mL with 100 mM sodium acetate buffer (pH 4.5). Then, aliquots of this solution were incubated with AMG solution for 20 min at 50 °C. After the addition of the GOPOD reagent, all samples’ solutions were incubated for more than 20 min, and then the content of non-resistant starch was calculated by measuring the absorbance at 510 nm. Total starch content is the sum of both (RS and non-RS).

### 3.3. In Vitro Digestion of Total Dietary Fiber (TDF) 

TDF was determined using the method of Prosky et al. [99]. To eliminate both protein and starch, samples were gelatinized using heat-stable amylase (pH 6.0, 95 °C, 30 min) followed by being sequentially digested via protease (pH 7.5, 60 °C, 30 min) and amyloglucosidase (pH 4.5, 60 °C, 30 min). Using ethanol, TDF was precipitated, and then the remaining material was weighed following rinsing via acetone and ethanol along with drying. Corrections were made for protein and ash calculations.

### 3.4. Phenolic Compounds’ Extraction

The method of Peng et al. [100] with adjustments was utilized to extract free phenolic compounds from banana samples. To obtain a concentration of 80%, two grams of banana flour were combined with twenty milliliters of ethanol diluted with Milli-Q water in triplicate. The samples were then put in a shaker (ZWYR-240 incubator shaker, Labwit, Ashwood, VIC, Australia) for 16 h at 20 °C and 150 rpm to allow for the potential release of phenolic compounds. The samples were centrifuged at 8000× *g* rpm for 15 min (ROTINA380R, Hettich Refrigerated Centrifuge, Tuttlingen, Baden-Württemberg, Germany). The supernatant was purified with a 0.45 m syringe filter and stored at −20 °C for further study.

### 3.5. In Vitro Gastrointestinal Digestion

In accordance with Gu et al. [94], the gastrointestinal digestion of green banana flour samples was performed with a minor modification of the oral, gastric, and intestinal phases using simulated oral (SOF), gastric (SGF), and intestinal (SIF) fluids based on static in vitro settings adhering to the harmonized INFOGEST 2.0 regulations. Water was blended with powder samples at a ratio of 2:1 (*v*/*w*), and a 5 mL aliquot of the mixture was obtained without digestion. SOF (1:1, *v*/*v*) was then added to the mixture to modify the pH to approximately 7.0, preceding a further addition of 75 U/mL of salivary amylase and 2 min of incubation at 37 °C with continued stirring. Five mL was then collected as the oral stage aliquot. The gastric phase was mimicked by adding SGF (1:1, *v*/*v*) and 2000 U/mL porcine pepsin to the oral bolus. The pH of the mixture was reduced to approximately 3.0 by adding HCL and incubating for 2 h at 37 °C. The aliquot of gastric digestion was obtained at 5 mL, and the digestion ended by restoring pH to 7.0. To initiate intestinal digestion, SIF (1:1, *v*/*v*), 100 U/mg trypsin, and 10 mM bile salt were incorporated into the gastric aliquot before incubation with the same conditions as before. Following incubation, aliquots of every digestion phase were frozen with liquid nitrogen to halt the enzymatic reactions.

### 3.6. In Vitro Colonic Fermentation

In accordance with Gu et al. [94], the non-absorbed portion after digestion in the gastrointestinal tract was continuously fermented with adjustments in the environment of human gut microbiota. As a replacement for human feces, pig feces were obtained from ten combined male and female finisher white pigs (50 kg live weight) raised in the animal house of Diamond Valley Pork (Thomas Road, Laverton North, VIC, Australia) and fed standard meals for two weeks. An anaerobic container was used to combine and mix samples of fresh feces. The suspension (20% stool) was made by homogenizing 20 g stool with 80 g sterilized phosphate buffer (pH 7.0) for five minutes in a stomacher blender (MiniMix^®^ Lab Blender, Thomas Scientific, Logan Township, NJ, USA). The blended fecal medium was passed through a sterile layer of muslin and then became usable. The intestinal digested food particle was then combined with 5 mL of fecal mixture and basal media in 6 batches of tubes that were flushed with nitrogen. Six groups of test tubes were agitated at 120 rpm and incubated in the dark for 0, 3, 6, 12, 24, and 48 h, respectively. The supernatant was obtained after centrifugation for 10 min at 10,000× *g* and 5 °C and was then prepared to evaluate phytochemical biological activity and generation of SCFAs.

### 3.7. Estimation of Phenolic Content and Antioxidant Capacity

#### 3.7.1. Total Phenolic Content (TPC)

The total phenolic content of banana samples was estimated according to Mussatto et al. [101] with some adjustments. In brief, sample extract or gallic acid standard (0–200 g/mL), Folin–Ciocalteu’s reagent solution, and water (1:1:8, *v*/*v*/*v*) were sequentially added to a 96-well plate, followed by a 5 min incubation at 25 °C in a darkened area. Ten percent (*w*/*w*) sodium carbonate was mixed with the same amount of sample and the identical circumstances were maintained for a further one hour of incubation. The absorbance was detected at 765 nm using a UV–VIS spectrophotometer (Thermo Fisher Scientific, Waltham, MA, USA) manufactured by Thermo Fisher Scientific. The findings of three independent assays were reported as mean mg gallic acid equivalents (GAE)/gram depending on dry weight (mg GAE/g) ± standard deviation (SD).

#### 3.7.2. Total Flavonoid Content (TFC)

The total flavonoid content of banana flour samples was determined using the approach of Suleria et al. [102] after some modifications. In summary, sample extract or quercetin standard (0–50 g/mL), 2% aluminum chloride, and 50 g/L sodium acetate (1:1:1.5, *v*/*v*/*v*) were sequentially administered to a 96-well plate and then subjected to a 2.5 h incubation at 25 °C in a dark place. At 440 nm, the absorbance was obtained. The outcomes of three independent tests were represented as the mean mg of quercetin equivalents/gram of dry mass (mg QE/g) ± standard deviation (SD).

#### 3.7.3. Total Condensed Tannins (TTC)

According to Suleria et al., the total tannin contents of banana samples were analyzed. Briefly, sample extract or catechin standard (0–1 mg/mL), vanillin solution, and 32% sulfuric acid (1:6:1, *v*/*v*/*v*) were sequentially added to a 96-well plate, immediately followed by 15 min of incubation at 25 °C in the dark. At 500 nm, the absorbance was calculated. The outcomes of three independent assays were presented as the mean mg catechin equivalents (CE) per dry weight (mg CE/g) ± (SD).

#### 3.7.4. 2,2′-Diphenyl-2-picryl-hydrazyl (DPPH)

Using the methodology of Suleria et al., the free radical scavenging of green banana samples was evaluated. Mixing DPPH dye and methanol (1:25, *w*/*w*) produced the 0.1 mM DPPH radical solution. The experiment was performed by adding a 40 μL sample extract or Trolox standard (0–200 g/mL) and 260 μL of formulated DPPH solution to a 96-well plate, then incubating at 25 °C for 30 min. At 517 nm, the absorbance was determined. The results of three independent assays were reported as the mean mg of Trolox equivalents (TE) per gram of dry mass (mg TE/g) ± (SD).

#### 3.7.5. Ferric Reducing Antioxidant Power (FRAP)

The banana FRAP analysis was elucidated by Suleria et al.’s study. In the dark, the FRAP dye solution was made by combining 300 mM sodium acetate, 10 mM TPTZ solution, and 20 mM Fe [III] solution in the proportion of 10:1:1 (*v*/*v*/*v*). In brief, 20 μL of sample extract or Trolox standard (0–200 g/mL) and 280 μL of dye solution were added to a 96-well plate, then incubated for 10 min at 37 °C. At 593 nm, the absorbance was obtained. The findings of three independent assays were reported as the mean mg of Trolox equivalent (TE)/gram of dry weight (mg TE/g) ± standard deviation.

### 3.8. Quantification of Phenolic Compounds via High-Performance Liquid Chromatography Photodiode Array (HPLC-PDA)

The targeted phenolic compounds in green banana flour were quantified using Agilent 1200 series HPLC (Agilent Technologies, Santa Clara, CA, USA) coupled with a photodiode array (PDA) reader by the previous published protocol of Suleria et al. with a few adjustments. A 0.45 µm syringe filter (PVDF, Millipore, MA, USA) was utilized to filter sample extracts. A Synergi Hydro-RP column (250 4.6 mm i.d.) with a 4 µm particle size (Phenomenex, Lane Cove, NSW, Australia) was guarded by a Phenomenex C18 ODS guard column (4.0 × 2.0 mm i.d.). The volume of injection of samples or standards was 20 µL. The mobile phases A and B were composed of water/acetic acid (98:2, *v*/*v*) and acetonitrile/water/acetic acid (55:43:2, *v*/*v*/*v*). The range of gradient profile was 10–25% B (0–20 min), 25–35% B (20–30 min), 35–40% B (30–40 min), 40–55% B (40–70 min), 55–80% B (70–75 min), 80–90% B (75–77 min), 90–100% B (77–79 min), 100–10% B (79–82 min), isocratic 10% B (82–85 min). The flow rate was 0.8 mL/min, and the column temperature was ambient. At the PDA detector, the wavelengths 280, 320, and 370 nm were chosen at the same time. The (2010) version of Empower 3 Software was utilized for device management, gathering data, and chromatography analysis.

### 3.9. Gastrointestinal Digestion and Colonic Fermentation Parameters

#### 3.9.1. Bioaccessibility of Phenolic Compounds

Every phenolic component’s bioaccessibility was calculated as the quantity of compound liberated from samples in every digestion stage and accumulated in the residual part over the in vitro digestion process of the GI tract and gut fermentation. The following formula was used to calculate bioaccessibility:

Oral Bioaccessibility (%) = (Oral portion/Total phenolic amount) × 100.

Gastric Bioaccessibility (%) = (Gastric portion/Total phenolic amount) × 100.

Intestinal Bioaccessibility (%) = (Intestinal portion/Total phenolic amount) × 100.

Colonic Bioaccessibility (%) = (Colonic portion/Total phenolic amount) × 100.

#### 3.9.2. Phenolic Recovery Index

The fractions of soluble as well as insoluble phenolics of the entire banana digesta were employed to calculate the recovery rate using the following formulas:

Soluble phenolics (%) = (Content in soluble fraction/Total phenolic content) × 100.

Insoluble phenolics (%) = (Content in insoluble fraction/Total phenolic content) × 100.

Recovery Index (%) = Soluble (%) + Insoluble (%).

#### 3.9.3. Residual Colonic Digesta Index

Residual phenolic compounds of colonic digesta (RCD%) is the non-bioavailable proportion after colonic fermentation, as computed by the following formula:

RID (%) = [Colonic insoluble fraction/Total phenolic content] × 100.

### 3.10. Evaluation of Short-Chain Fatty Acids (SCFAs)

The analysis of SCFAs was carried out with a minor modification to the previous methodology of Gu et al. [94]. One gram of each phase of bowel fermentation was combined with five milliliters of water and vortexed for three minutes. The samples were then acidified with 5 mol/L HCL to a pH of approximately 2.0, followed by 10 min of centrifugation at 10,000× *g* rpm and 10 °C. A quantity of 1 mL of sample supernatant was added to 4 mL of diluted acid mix that consisted of 1% formic acid and 1% orthophosphoric acid, and then mixed thoroughly. The 4-methyl-valeric solution with an overall concentration of 1.59 mmol/L was used as an internal standard. Analytical standard curves were created using acetic, propionic, butyric, iso-butyric, and valeric acids. All prepared chemicals and standards were kept at 4 °C and were made for SCFA evaluation. The colon digesta were analyzed for SCFAs using gas chromatography (7890B Agilent, Santa Clara, CA, USA) equipped with a flame ionization detector (GC-FID), an autosampler (Gilson GX-271, Gilson Inc., Middleton, WI, USA) and autoinjector. For the estimation of SCFAs amounts, we used a capillary column (SGE BP21, 12 × 0.53 nm internal diameter with 0.5 µm film thickness, SGE International, Ringwood, VIC, Australia, P/N 054473) with retention gap kit (including a 2 × 0.53 mm ID guard column, P/N SGE RGK2). The volume of the injection was 1 µL. The carrier gas was helium with a total flow rate of 14.4 mL/min, and the composition gas was composed of nitrogen, hydrogen, and air with respective flow rates of 20, 30, and 300 mL/min. The oven temperature was set to 100 °C for 30 s, then raised to 180 °C for 1 min at a rate of 6 °C/min, and then retained at 200 °C for 10 min at a rate of 20 °C/min. The detector and injection port temperatures were set to 240 and 200 °C, respectively. For subsequent statistical analysis, all results were expressed in mmol/L.

### 3.11. Statistics Analysis

The outcomes of this study were given as the means ± standard deviation (SD) of three independent evaluations using nine technical replicates and three biological replicates. One-way analysis of variation (ANOVA) and Tukey testing were conducted to confirm the statistical significance difference between the three green banana cultivars and the digestion levels with respect to the phenolic content, antioxidant capacity, dietary fiber, and resistant, non-resistant, and total starch (*p* ≤ 0.05). Minitab 19 (Minitab^®^ for Windows Release 19, Minitab Inc., Chicago, IL, USA) and GraphPad Prism 10 (Prism 10.0.2, GraphPad Software Inc., San Diego, CA, USA) were used for the data analysis and graph formations. All presented findings have been corrected with blank or control values.

## 4. Conclusions

In summary, the improvement of phenolic compounds’ bioaccessibility in green bananas and their antioxidant properties can be achieved through the processes of in vitro digestion and colonic fermentation, assisted by the digestive enzymes and gut microbiota. In general, variations in banana cultivars can impact the bioaccessibility of individual phenolic compounds, and the intestinal phase yields the highest bioaccessibility for most of these compounds. After 48 h of colonic fermentation, phenolic compounds’ bioaccessibility is still significantly detectable. The antioxidant capacity of digested bananas can potentially be retained or enhanced through the subsequent breakdown of complex phenolic compounds. Unripe bananas could provide considerable concentrations of SCFAs over 24 h fecal reactions, which can be attributed to their high RS and TDF content levels. Therefore, based on the current project, the substantial antioxidant potential and high amount of dietary fiber discovered in an unripe banana would potentially enhance gut health upon ingestion. Nevertheless, additional research is necessary to fully understand the precise metabolic conversion of phenolic compounds due to the significant presence of indigestible dietary fibers and starch fractions in bananas.

## Figures and Tables

**Table 1:** Digestibility of starch and dietary fiber of whole green banana.

Samples	N-RS% (g/100 g)	RS% (g/100 g)	TS% (g/100 g)	TDF% (g/100 g)
Cavendish	23.39 ± 0.13 ^a^	31.46 ± 0.17 ^c^	54.85 ± 0.11 ^b^	31.28 ± 0.38 ^b^
Ladyfinger	11.89 ± 0.18 ^c^	48.99 ± 0.21 ^a^	60.88 ± 0.04 ^a^	31.01 ± 0.66 ^b^
Ducasse	16.22 ± 0.20 ^b^	35.22 ± 0.08 ^b^	51.44 ± 0.12 ^c^	38.72 ± 0.25 ^a^

Digestibility of starch and dietary fibers of whole green banana. The results of all assays were expressed as mean ± standard deviation (*n* = 3) on dry weight basis. Values within the same column with different superscript letters (^a–c^) are significantly different. RS: resistant starch, N-RS: non-resistant starch, TS: total starch, TDF: total dietary fiber.

**Table 2:** Estimation of bioaccessibility after in vitro digestion and colonic fermentation for phenolic compounds of green banana.

No	Compound	Oral BIA (%)			Gastric BIA (%)			Intestinal BIA (%)			Colonic BIA (%)		
		Cavendish	Ladyfinger	Ducasse	Cavendish	Ladyfinger	Ducasse	Cavendish	Ladyfinger	Ducasse	Cavendish	Ladyfinger	Ducasse
1	Gallic acid	27.63	26.98	28.96	29.08	36.19	39.58	79.11	68.99	41.50	5.56	6.75	8.25
2	Protocatechuic acid	30.15	32.67	30.57	40.35	43.68	56.94	54.41	61.26	36.67	6.99	6.67	8.11
3	Caftaric acid	0.00	0.00	0.00	47.62	46.79	49.24	52.38	53.21	57.06	10.65	11.63	0.00
4	P-hydroxybenzoic acid	28.87	31.66	28.87	32.45	40.31	36.51	45.82	37.24	42.41	6.73	8.19	6.95
5	Catechin	27.54	26.62	27.39	51.55	58.57	50.69	71.56	64.59	30.21	6.69	8.15	0.00
6	Chlorogenic acid	33.24	32.84	30.79	38.45	41.38	54.54	49.85	48.43	55.50	8.66	0.00	12.66
7	Caffeic acid	0.00	0.00	27.07	49.99	48.17	33.23	51.60	50.35	25.69	5.23	6.30	7.64
8	Syringic acid	27.34	30.32	29.14	33.18	36.15	32.77	50.84	39.68	34.30	4.76	5.77	0.00
9	Epicatechin	33.09	32.49	32.63	40.70	47.76	55.47	51.62	35.17	52.27	0.00	0.00	0.00
10	Coumaric acid	22.27	24.03	25.68	33.25	33.29	50.72	47.25	25.14	57.68	4.80	5.77	7.01
11	polydatin	26.03	21.57	24.82	34.91	30.55	40.06	43.39	33.44	36.76	1.23	0.00	0.00
12	Diosmin	15.79	26.45	17.20	27.76	32.72	24.79	38.51	35.99	27.30	0.13	0.25	0.18
13	Resveratrol	32.92	31.20	30.90	40.67	41.51	48.35	57.78	19.12	49.76	4.92	0.00	0.00
14	Quercetin	37.60	36.72	33.26	44.14	42.74	40.25	53.48	30.86	42.42	33.66	40.51	49.21
15	Kaempferol	20.62	13.71	22.09	34.75	33.31	36.46	33.45	28.67	35.92	0.00	0.00	0.00
	Total phenolic compounds	27.91	26.06	27.53	40.41	41.17	43.45	51.26	38.72	42.24	22.08	18.18	15.34

**Table 3:** Estimation of recovery (%) and residual digesta index (RCDI) (%) after colonic fermentation for phenolic compounds of green banana.

No	Compound	Colonic Recovery (%)			Residual Colonic Digesta Index (%)		
		Cavendish	Ladyfinger	Ducasse	Cavendish	Ladyfinger	Ducasse
1	Gallic acid	11.79	16.92	14.80	6.23	10.17	6.55
2	Protocatechuic acid	16.46	18.12	15.52	9.47	11.44	7.42
3	Caftaric acid	24.08	31.65	13.01	13.44	20.02	13.01
4	P-hydroxybenzoic acid	13.18	18.50	13.32	6.45	10.31	6.36
5	Catechin	15.69	8.15	9.03	9.00	0.00	9.03
6	Chlorogenic acid	20.43	18.45	24.40	11.77	18.45	11.74
7	Caffeic acid	5.23	6.30	14.69	0.00	0.00	7.04
8	Syringic acid	4.76	5.77	0.00	0.00	0.00	0.00
9	Epicatechin	11.05	16.77	0.00	11.05	16.77	0.00
10	Coumaric acid	4.80	5.77	7.01	0.00	0.00	0.00
11	polydatin	2.90	2.54	1.65	1.68	2.54	1.65
12	Diosmin	0.13	0.25	0.18	0.00	0.00	0.00
13	Resveratrol	4.92	10.29	6.71	0.00	10.29	6.71
14	Quercetin	33.66	40.51	49.21	0.00	0.00	0.00
15	Kaempferol	30.91	0.00	30.50	30.91	0.00	30.50
	Total phenolics	27.66	32.05	27.79	84.51	77.07	79.75

## Data Availability

The data presented in this study are available in this manuscript.
